# A Case of Penetrating Incised Wound of the Abdomen and Small Intestine, Resulting in Death from Internal Strangulation

**Published:** 1854-11

**Authors:** Edward Hartshorne


*A case of Penetrating Incised Wound of the Abdomen and Small Intestine, resulting in death from internal strangulation.* By EDWARD HARTSHORNE, M. D.

The following report has been drawn up from notes taken by the writer nearly twelve years ago, while serving in the Pennsylvania Hospital as house surgeon, under the superintendence of the late Dr. Randolph, who was then on duty as attending surgeon. It was thought to possess sufficient interest as an instance of peculiar homicidal injury, as well as uncommon termination of abdominal wound, to warrant its publication, even at this long interval, and notwithstanding the lack of clinical details to which we are condemned by the unfortunate meagreness of the only data now to be obtained.

Wm. Thomas, mulatto, aged 25, of rather slight frame but excellent previous health and temperate habits, was wounded in the abdomen with a sharp instrument, on the evening of the 13th of November, 1842.

He was brought to the Pennsylvania Hospital where he was seen by the resident physician within forty-five minutes after the reception of the wound.

When first examined in the ward he was lying on his back with his limbs extended, appeared to be entirely free from excitement, and exhibited no evidence of prostration or of any uneasiness, except some degree of pain and soreness in the injured part.

According to his own statement he chased his antagonist at least twenty paces, and struck him with a stick two or three times after the infliction of the injury; he then ran some two hundred yards to an apothecary shop, where he first discovered the protrusion of his bowel. From this shop he was immediately conveyed, in a cab, some four or five hundred yards, to the Hospital. The only sensation perceived at the moment when the knife entered his body, was that of slight nausea. The man had eaten an ordinary supper and taken a glass of beer some three hours previous to the occurrence.

Uncovering the patient brought into view, resting in front of the abdomen and protruding from a wound in its parietes, a pulsating mass, which consisted of two loops of small intestine with their adjoining mesentery. The upper of these loops was about eight, and the lower about twelve inches long; they were much darkened in color from venous engorgement, but clean, moist and glistening, and appeared to be half filled with semifluid matter. The lacteals in the mesentery were distended with a white fluid, and resembled in size, shape, color and abrupt termination, so many pieces of pack-thread.

The wound proved to be incised, an inch in length, and extending almost longitudinally downwards from about two inches and a half below and an inch to the left of the umbilicus, penetrating the rectus muscle in the direction of its fibres. Besides this, however, there existed on the outer surface of the most dependent portion of the lower loop, a transverse opening into its canal, at least eight lines in length, giving exit on pressure to alimentary matter mixed with blood, and presenting the clean edges, and characteristic everted mucous margins, of a penetrating incised wound of the intestines.

Notwithstanding the relaxation of the abdominal muscles by flexion of the thighs and elevation of the shoulders, the neck of the hernial tumor was so firmly constricted by the external wound, that safe reduction seemed impracticable without enlargement of the aperture. The incision in the abdominal wall was accordingly prolonged half an inch upwards, by means of a strait bistoury cutting from within outwards, and carefully guarded with a silver director, so as not to touch the peritoneum or other encroaching membranes. The stricture having been thus removed, the whole protruding bowel, except about two inches of its length, which included the wounded part, was gradually reduced. The intestinal wound, which, on account of its transverse direction and the protrusion of mucous lining, gave us little apprehension of extravasation, was now secured according to the plan of Jobert. This was effected with two sutures of fine waxed silk ligature, drawn through all the coats of the canal in such a manner as to bring into contact the outer surface of the peritoneal coat, along the whole length of the transverse opening, by turning in its edges like an ordinary seam. The ends of the ligatures were next cut off, and the wounded portion at once returned into the cavity. The closure of the external wound then remained to complete the dressing. Here, on account of the impossibility, in so small an opening, of suppressing the abdominal contents, which were constantly bulging up, it was out of the question, even if otherwise desirable, to pass a suture needle through the muscular parietes without incurring great risk of puncturing the intestine. The margins of integument alone, therefore, were approximated with three sutures and as many adhesive strips. A compress of lint was lastly applied over the wound and confined with a circular bandage, so as to prevent hernial protrusion, and enable the longitudinal muscular fibres to approach and unite with each other, if possible, by the first intention.

An opiate of sixty drops of laudanum was given to the patient before the dressing; and although the handling of the exposed parts, unavoidable in their replacement, appeared to occasion a good deal of pain, yet the operation was followed by immediate relief.

Not more than eight ounces of blood had been lost externally, and the only symptom apparent when the man was left for the night, was some degree of soreness of the wound on pressure.

On the following day, November 14th, he presented the following condition. Pulse 96, pretty full and strong, with some head-ache, heat of skin and furred tongue; had slept but little through the night. Very slight meteorism with soreness on pressure around the wound. Bladder has been evacuated in bed without difficulty. No stool. He was bled twenty-four ounces, and directed to take Ant. et Pot. Tart. gr. 1-12th, in Mixt. Neutral. f.℥ss. q. h., and Cal. gr. ss., P. Opii gr. j., q. h. t., during the day. Barley water diet. The bleeding removed the head-ache, and reduced his pulse in force and frequency. In the evening, the fever and pain were gone ; meteorism no greater, and soreness around the wound unchanged. Omit the diaphoretic mixture and suspend the cal. and op.

November 15th, A. M.—Patient has slept well throughout the night, and is in good spirits. Pulse 84, soft, skin cool, expression of face good, tongue moist and slightly furred. Tympanitis somewhat increased, with slight pain on pressure over abdomen. He began to complain of violent pain in his bowels about noon of this day, at the same time that the tympanitis and tenderness were very much increased. The bandage was loosened, and Morph. Sulph. gr. ss. immediately administered ; eight ounces of blood were taken from his arm, until he nearly fainted. The calomel and opium were directed to be given every two hours. Under the influence of the opiates and venesection, the pain moderated. In the evening, the tympanitis was found to be undiminished, and produced great discomfort, particularly in the upper half of the abdomen, which was much more distended than the lower. The pain, also, had again become violent. Ordered Cal. gr. iij. P. Opii. gr. ij. at once, the pills of cal. and op. to be resumed in two hours, and repeated every hour until patient is relieved or decidedly narcotised. Enemata of Ol. Terebinth. in castile soap suds were administered three times in the course of the night, and afforded relief by the expulsion of large amounts of flatus. A single ten grain dose of assafœtida was exhibited in the hope of its relieving the stomach and upper intestines, but it only created nausea and vomiting with hiccough and eructation.

Wednesday 16th, A. M.—Tympanitis still increasing, and now produces very great oppression. Tenderness on pressure, also, aggravated, especially just above the wound ; although the latter looks remarkably well, and presents no evidence of protrusion. Countenance becoming anxious ; nausea, hiccough and vomiting more frequent, and pulse more decidedly depressed ; leeches, warm fomentations and anodyne enemata were successively employed to no material purpose.

Stercoraceous vomiting made its appearance on the fifth day, and the distension, pain, singultus and other symptoms of internal strangulation continued to increase in violence, until the patient finally succumbed on the morning of December 20th, six days and six hours after the reception of the wound.

*Autopsy eleven hours after death.* The external aspect presented nothing worthy of especial note except the very great distension of the upper two-thirds of the abdominal region. The wound in the integuments had cicatrized completely without any hernial protrusion.

In order to expose the abdominal contents as nearly as possible in situ and without disturbing the relations of the wounded and otherwise altered parts, an oval incision was made through the parietes of this cavity along its costal margin above, down each side and along the pelvic margin below. The portion of abdominal wall thus detached was next carefully turned over at its circumference so as to enable us to look beneath. We then ascertained the cause of strangulation to be an accidental overlapping of a knuckle of one of the hernial loops of small intestine over another portion of the same loop, the overlapping portion being closely bound down upon the other by bands of adhesive membrane, through which it had become attached to the parietal peritoneum in front.

The constriction thus produced was at least half an inch in length, and amounted to a complete occlusion of the alimentary canal. The small intestine was excessively distended as well as externally injected, and at the same time partially adherent in three or four transverse folds, some two-thirds of its length down to the seat of stricture. No distension present below this point. Inflammatory action nowhere very strongly marked, although more so than elsewhere across the whole cavity from two to three inches above the umbilicus down to within about three inches of the pubis. The folds of small intestine and the omentum occupying this region were slightly adherent together and to the peritoneum, and were studded with small patches of false membrane, which appeared to be more numerous and firm on the left side and about the wound, but by no means in such excess as to denote a fatal amount of peritonitis. The wounded part of the bowel was found entirely closed and firmly attached to the peritoneum about three inches above and one inch to the left of the external wound. No effusion of alimentary contents had taken place, nor had any undue inflammation been excited immediately around this spot. Upon separating the newly formed adhesions we discovered the sutures fastened to the inner surface of the peritoneum, and within the intestinal canal, into which they appeared to have just finished working their way. The wound in the rectus muscle appeared to have united, although some extravasated blood yet remained unabsorbed. No effusion of serum, and no separate flakes of lymph or traces of pus could be detected. Abdominal peritoneum slightly mapped with fine arborescent injections, but not thickened or roughened except in the parts adjoining the folds and points of adherent bowel already described. Elsewhere the evidence of inflammation was unimportant. Other organs of the body in each of the cavities were examined in detail and found to be in perfect health.

The case presented in the foregoing sketch is interesting in many respects, but chiefly so on account of its termination in fatal incarceration or internal strangulation. This accident seems to be of rare occurrence, judging from the small number of analogous facts on record. It is certainly one of great importance in many of its relations, especially where the primary lesion is, as in the present instance, the result of criminal violence. The man who inflicted the original injury of which our patient was apparently the victim, was subsequently tried for his life upon the charge, and was very glad to escape with a long imprisonment for murder in the second degree. The medico-legal aspects of the case, together with some other considerations suggested by it, will form the subject of another communication, in which the writer hopes to present a resumé of a number of the published cases of similar character. He regrets that since the inquiry was suggested by the re-perusal of the notes upon which his own report is based, he has not yet found time to complete the extended references required for such a purpose.

